# First Reported Case of Simultaneous Transjugular Tricuspid Valve-in-Ring and Pulmonary Valve-in-Valve Implantation

**DOI:** 10.1016/j.jaccas.2025.105931

**Published:** 2025-11-04

**Authors:** Mohamed Al Nasef, Bandar Al Shehri, Ahmed Al Zahrani, Merna Atiyah, Saria Mansour Ahmed, Khalid Al Najashi

**Affiliations:** a Prince Sultan Cardiac Center, Riyadh, Saudi Arabia; b King Hamd American Mission Hospital, Mayo Clinic Care Network Hospital, Manama, Bahrain; c Suliman Al Habib Hospital, Khobar, Saudi Arabia

**Keywords:** tetralogy of Fallot, tricuspid valve, pulmonic valve

## Abstract

**Background:**

Patients with repaired tetralogy of Fallot often require reinterventions for right-sided valve dysfunction. Surgical reoperations carry high morbidity, making transcatheter valve-in-valve and valve-in-ring procedures valuable alternatives.

**Case Summary:**

A 46-year-old man with repaired tetralogy of Fallot, prior tricuspid annuloplasty, pulmonary bioprosthesis, and transcatheter pacemaker presented with advanced right heart failure. Imaging showed massive right atrial enlargement, severe tricuspid stenosis/regurgitation, pulmonary dysfunction, and indwelling leads, precluding transfemoral access. Repeat surgery was prohibitive; thus, simultaneous transcatheter intervention was pursued. Via a transjugular approach, Edwards Sapien valves were implanted in both the tricuspid and pulmonary positions, with complete resolution of regurgitation.

**Discussion:**

This case illustrates the feasibility of dual transjugular right-sided valve replacement in complex congenital heart disease. Meticulous imaging and planning allowed safe navigation of anatomical challenges.

**Take-Home Message:**

A combined transjugular-approach valve-in-ring and valve-in-valve procedure can provide an effective alternative when surgery is prohibitive.

## Visual Summary

**Figure:**
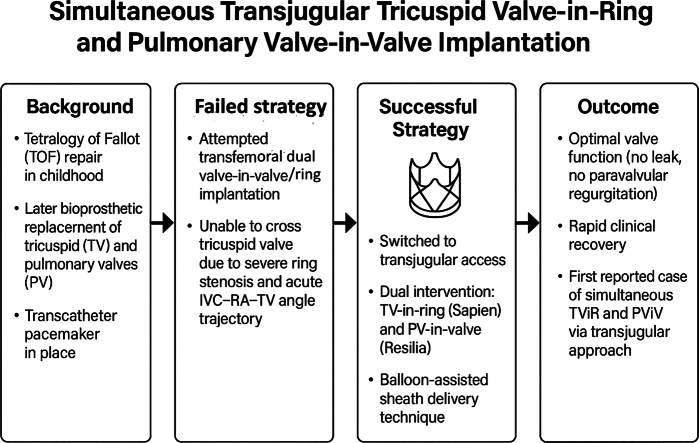

Right-sided bioprosthetic valve dysfunction involving the tricuspid and pulmonary positions is a recognized late complication in adults with repaired congenital heart disease, particularly tetralogy of Fallot (ToF). Surgical reintervention in this population carries significant risks, especially in patients with multiple prior sternotomies, pacemaker dependency, and end-organ dysfunction.^1^ In recent years, transcatheter valve-in-valve (ViV) and valve-in-ring (ViR) procedures have emerged as less invasive alternatives to redo surgery, offering meaningful symptomatic relief with reduced procedural morbidity.^1^

**Take-Home Messages**

• Massive right atrial enlargement may preclude transfemoral access, favoring the transjugular approach.
• Combined transcatheter valve-in-ring and valve-in-valve procedures can be safe and effective when surgery is prohibitive.

The transjugular approach has been increasingly used for right-sided structural valve interventions given its more direct and stable alignment with the tricuspid and pulmonary annuli, especially in patients with challenging inferior vena cava (IVC)–right atrium–tricuspid valve angulation. This access route is particularly advantageous in patients with massively dilated right atria, where transfemoral navigation can be technically prohibitive.^2^

Here, we present a case of simultaneous transjugular tricuspid ViR and pulmonary ViV implantation, performed in a patient with severe bioprosthetic degeneration and prohibitive surgical risk. Although isolated transcatheter ViV or ViR procedures in the tricuspid or pulmonary position have been reported, simultaneous dual-valve implantation via a transjugular approach has not previously been reported. Furthermore, use of the Edwards Resilia valve in the pulmonary position through this route is, to our knowledge, unreported in the literature.

## Case Presentation

A 46-year-old man with repaired ToF has had a lifelong history of complex cardiac interventions. In infancy, he underwent a right Blalock-Taussig shunt, followed by complete ToF repair at age 5 in 1983. Postoperatively, he developed an infected patch, resulting in a flail tricuspid valve and residual ventricular septal defect, which were subsequently managed at age 6 with tricuspid annuloplasty, ventricular septal defect closure, and implantation of a single-lead right ventricular (RV) pacemaker, later revised to a transvenous system. In his early twenties, the patient experienced a cerebrovascular accident complicated by intracranial hemorrhage and resultant right-sided weakness. In 2001, at age 31, he underwent pulmonary valve replacement with a 25-mm Carpentier-Edwards bioprosthesis. After a prolonged loss to follow-up, he re-presented 3 months ago in NYHA functional class III and IV with cachexia, peripheral edema, abdominal distension, ascites, and complications of right-sided heart failure and lower-limb cellulitis, managed medically. Physical examination revealed signs of chronic right-sided congestion—jugular venous distension, prominent abdominal wall veins, hepatomegaly—and laboratory evaluation confirmed hepatic dysfunction. The right atrium constituted more than 60% of the cardiac silhouette.

**Figure 1:**
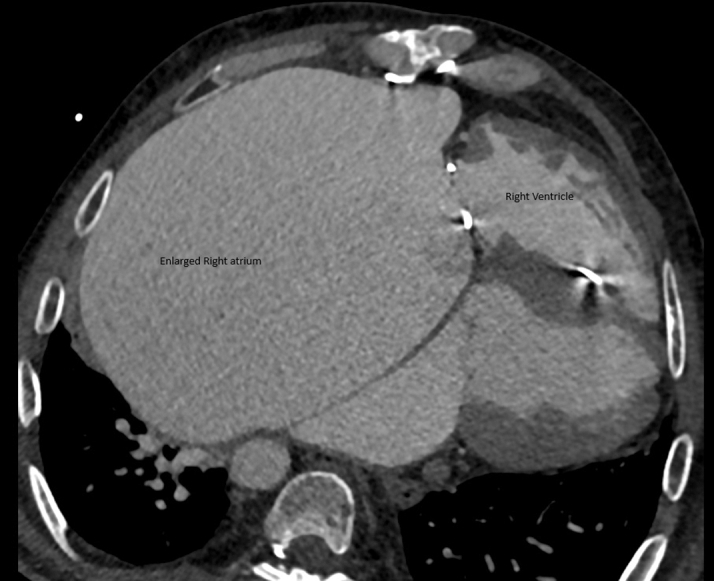
Cardiac Computed Tomography Axial Cut Demonstrating Markedly Enlarged Right Atrium

**Figure 2:**
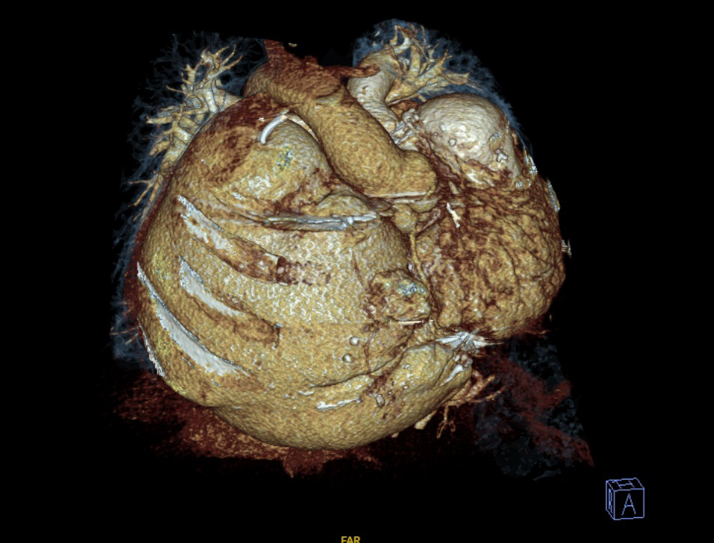
Cardiac Computed Tomography Three-Dimensional Reconstruction Showing a Markedly Dilated Right Atrium

**Figure 3:**
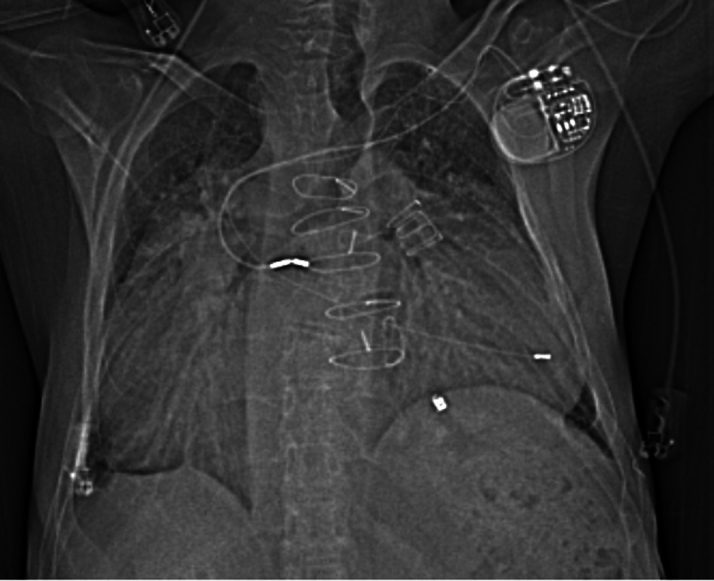
Topogram of the Chest Showing Massive Right Atrial Enlargement

Preintervention transthoracic echocardiography demonstrated severe tricuspid stenosis and regurgitation, severe pulmonary regurgitation with moderate stenosis (mean transtricuspid gradient: 20 mm Hg, peak pulmonary transprosthetic gradient: 60 mm Hg), massive right atrial dilation occupying over 60% of the cardiac silhouette as demonstrated by cardiac computed tomography (CT) (Figures 1, 2, and 3), enlarged IVC, and impaired RV function with low to normal left ventricular performance. Preprocedural CT showed severe tricuspid annular narrowing, with an annular area of approximately 4.9 cm^2^ and perimeter-derived diameter of 26.1 mm.

Given the patient's pacemaker dependence, multiple prior sternotomies, decompensated heart failure, and hepatic impairment, surgical reintervention posed a prohibitively high risk. Multidisciplinary evaluation—including consultation with Edwards medical specialists—led to the decision for a transcatheter approach, to address both the pulmonary and tricuspid valves. This strategy aligns with emerging evidence supporting concomitant intervention in patients with severe valvular dysfunction. The patient and family were extensively counseled regarding the risks, benefits, and alternative options, and after thorough discussion, informed consent was obtained with active participation in the decision-making process given the high-risk and complex nature of the case.

## Procedural Course

### Attempted transfemoral approach

An initial attempt to perform the procedure via the transfemoral venous route was undertaken but proved technically unfeasible owing to extreme anatomical distortion. Multiple catheter systems were employed in an effort to cross the degenerated tricuspid valve ring, including a Berman wedge catheter, Agilis steerable deflectable sheath, cut pigtail catheter, and Judkins right (JR) special catheter. Despite various wire and curve configurations, all attempts to cross the tricuspid ring were unsuccessful.

The procedure was challenged by a severely dilated right atrium and marked stenosis of the surgical tricuspid ring. The atrial enlargement significantly impaired catheter maneuverability and torque control. The usual technique of advancing across the tricuspid annulus using the atrial curvature of the catheter failed, as the lateral wall of the right atrium offered no effective countersupport, resulting in repeated prolapse of the catheters without engaging the annulus. Additionally, the acute angulation between the IVC and the tricuspid valve plane further limited coaxial alignment and directional control.

After prolonged attempts, the procedure was aborted to avoid excessive procedural time and patient instability, and the decision was made to proceed electively via the transjugular approach at a later date.

### Transjugular approach

One week after the failed transfemoral attempt, the procedure was reattempted via the right internal jugular vein, under general anesthesia with fluoroscopic and transesophageal echocardiography (TEE) guidance. A 14-F sheath was placed through the right internal jugular access, and full hemodynamic monitoring and pacing backup were ensured. Dedicated coronary compression testing was not undertaken, as preprocedural cardiac CT angiography demonstrated that the coronary arteries were well separated from the bioprosthetic valves, thereby excluding the possibility of compression upon valve deployment.

The right internal jugular approach significantly improved catheter trajectory, allowing near-coaxial alignment with the tricuspid annulus. A JR diagnostic catheter with a Terumo soft-tip wire was used to cross the stenotic Duran annuloplasty ring. Using this approach, the angle to cross the tricuspid ring was more feasible. Once the wire was across the annulus, it was carefully advanced and parked in the right pulmonary artery (RPA) to provide stable distal support. This was exchanged over a Terumo exchange to a long end-hole, stiffer JR catheter or guide catheter, which offered enhanced support and directional control. Over this, a backup Meier wire was successfully introduced and positioned. Based on prior procedural experience, the use of a longer, stiffer diagnostic or guiding JR catheter was found to be more effective during Meier wire advancement, as it helped prevent catheter prolapse back into the right atrium—a frequent challenge in patients with severely enlarged right atria and distorted geometry between the annulus and IVC.

**Figure 4:**
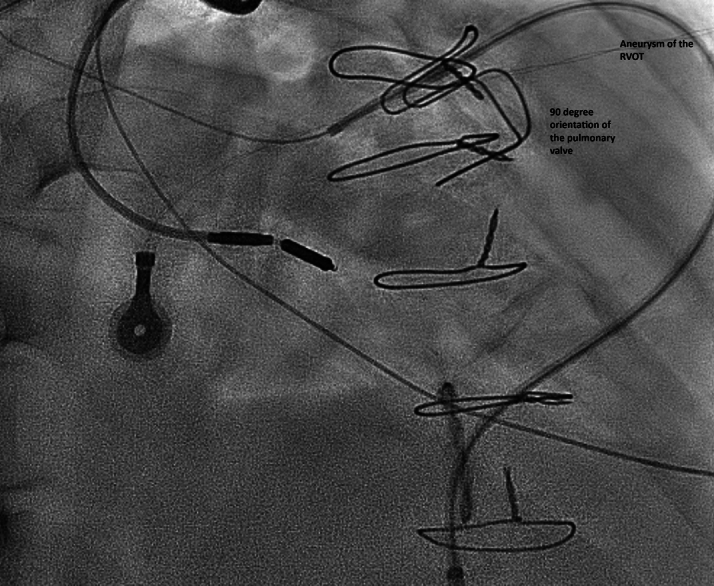
Extensive Cardiomegaly With a Markedly Enlarged Right Atrium Occupying a Significant Portion of the Cardiac Silhouette, Demonstrating Acute Angulation of the Existing Bioprosthetic Pulmonary Valve RVOT = right ventricular outflow tract.

Importantly, the previously implanted pulmonary bioprosthesis was positioned at a sharp 90° angle relative to the main pulmonary artery, with its valve opening directly facing the origin of the RPA (Figure 4). This anatomy created a steep angle between the main pulmonary artery and RPA and, in combination with the narrow tricuspid ring, made advancement of catheters technically challenging. Additionally, the presence of severe pulmonary regurgitation hindered catheter stability, precluding parking of the catheter in a controlled fashion. To overcome this, and to navigate the acute angle of the pulmonary valve, severe regurgitation, and RV outflow tract aneurysm—which caused catheter prolapse while advancing the wire—we exchanged the JR special catheter with a long JR end-hole catheter. Its enhanced stability prevented prolapse during advancement of the stiff exchange-length wire.

After wire placement into the RPA, before implantation of the pulmonary valve, balloon dilation of the surgical pulmonary bioprosthesis was performed using a size 20 Atlas balloon. This served a dual purpose: 1) to facilitate easier advancement of the large-bore sheath through the stenotic valve; and 2) to provide a visual and tactile assessment of the internal diameter to aid in selecting the appropriately sized transcatheter valve. This preparatory step contributed to smoother delivery system passage. Accordingly, we decided to implant a 23-mm Edwards Sapien S3 Ultra in the pulmonary position, as per the balloon sizing of the surgical prosthetic valve. Next, we proceeded to introduce a 24-mm DrySeal sheath. However, the standard dilator of the DrySeal proved challenging to advance across the stenotic tricuspid valve, as its stiffness and alignment repeatedly caused it to pull the entire system—including the sheath and wire—into the dilated hepatic veins. This repeated prolapse prevented controlled sheath advancement and compromised catheter trajectory.

To overcome this, the dilator was removed, and a novel technique was employed: An 8 × 80 mm–long peripheral angioplasty balloon was introduced, with half of the balloon positioned within the sheath and the other half extending over the wire beyond the tricuspid ring. The balloon was gently inflated, and with slow, controlled deflation, the sheath was advanced over the collapsing balloon across the tricuspid valve and into the RPA. This method provided improved torquability and coaxial support, and eliminated prolapse into the hepatic venous system (Video 1, Video 2, Video 3, Video 4, Video 5, Video 6, Video 7). Although the Sapien S3 valve was initially intended for implantation in the pulmonary position, an unexpected issue with valve storage rendered it unusable. Consequently, a 23-mm Edwards Inspiris Resilia valve was selected as an off-label alternative for pulmonary ViV implantation. The Resilia valve was deployed using the standard balloon-expandable technique in the pulmonary position. Imaging confirmed proper positioning and excellent valve function (Video 8).

Attention was then returned to the tricuspid valve. A 26-mm Sapien valve was implanted in a ViR configuration under TEE and fluoroscopic guidance, with a 70:30 ratio toward the RV to ensure appropriate sealing between the skirt and the annuloplasty ring. The pacing lead remained undisturbed, and no paravalvular leak was observed (Video 9). Postprocedure TEE demonstrated no regurgitation after pulmonary ViV and tricuspid ViR deployment, as well as no evidence of paravalvular leak (Videos 10 and 11).

Fluoroscopy time for the procedure was 147 minutes. Given the deranged coagulation profile of the patient, no intravenous heparin was administered throughout the intervention. The patient was extubated in the catheterization laboratory and was transferred in stable condition. Clinical recovery was rapid, with improvement in functional capacity, resolution of systemic congestion, and normalization of liver function tests. Follow-up echocardiography confirmed excellent valve function in both positions.

## Discussion

This case illustrates the unique challenges encountered in patients with advanced heart failure, multiple prior sternotomies, dual right-sided valve disease, and longstanding pacemaker dependence. Such clinical and anatomical complexity significantly narrows therapeutic options and renders conventional surgical reintervention prohibitively high risk. In this context, the transjugular approach has emerged as a valuable alternative to traditional femoral access for right-sided structural heart interventions, particularly in anatomically complex cases, allowing simultaneous management of both pulmonary and tricuspid valves while avoiding the morbidity associated with repeat sternotomy. Its trajectory offers a more direct, coaxial alignment with the tricuspid annulus and pulmonary outflow tract, especially in patients with massively dilated right atria or distorted IVC angles, where femoral approaches prove challenging. This strategy not only reflects the growing role of transcatheter therapies in complex congenital and structural heart disease but also underscores the importance of individualized, multidisciplinary decision-making in achieving optimal outcomes.

In patients with repaired ToF, tricuspid regurgitation is frequently secondary to RV dilation and may improve after pulmonary valve replacement alone. Nevertheless, in selected patients with persistent or severe regurgitation, combined percutaneous strategies—such as transcatheter tricuspid repair (eg, TriClip) together with pulmonary valve replacement—represent a promising alternative to surgery. These approaches may provide the advantages of reduced procedural morbidity and avoidance of repeat sternotomy, although they remain limited by device availability, anatomical suitability, and the need for long-term outcome data compared with conventional surgical repair or replacement. In our case, the patient had not only severe regurgitation but also significant stenosis across the tricuspid valve ring, necessitating combined percutaneous management of both the pulmonary and tricuspid valves. Tricuspid ViR and ViV procedures via the transjugular route have been increasingly reported in patients with prior annuloplasty or prosthetic valve dysfunction. Several studies have demonstrated improved technical success and procedural safety with this approach in patients with large right atria or prior failed femoral attempts.^1^^,^^3^ Similarly, pulmonary ViV implantation using balloon-expandable valves via transjugular access has shown favorable hemodynamic and clinical outcomes.^4^^,^^5^

There have been several reports describing the simultaneous tricuspid ViV and pulmonary ViV through transfemoral approach.^5^ Despite these advances, the current report is the first to describe a simultaneous transjugular tricuspid ViR and pulmonary ViV implantation. The case highlights the feasibility of a dual-valve intervention via a single access site in a critically ill adult with complex congenital heart disease and prior surgical repairs.

The use of the transjugular route allowed for superior catheter stability, particularly for tricuspid valve crossing where femoral access was limited by the severe dilation and distortion of the right atrium. The novel balloon-assisted sheath delivery technique further addressed challenges in advancing equipment across a stenotic ring within an enlarged cardiac chamber. Although this technique has been demonstrated in various live cases, including structural heart conferences and interventional training platforms, it remains under-reported in the peer-reviewed literature (Video 2, Video 3, Video 4, Video 5). Our case reinforces its value in achieving controlled advancement of large-bore sheaths through tortuous or unsupported right heart anatomy, and it highlights the potential for broader documentation and adoption of this method in complex right-sided interventions. Moreover, the precise deployment of the valve with a 70:30 ratio toward the RV optimized sealing and minimized interference with the pacing lead.

An important consideration during tricuspid ViV or ViR procedures is the presence of a pre-existing pacemaker or defibrillator lead. According to available scientific evidence, patients undergoing ViR procedures represent a high-risk subgroup, with elevated morbidity and mortality rates.^6^ These risks are compounded in individuals with prior congenital heart repairs, pacing systems, or significant right heart dilation. Prior literature supports the feasibility of implanting balloon-expandable valves, such as the Sapien 3, within surgical tricuspid rings in the presence of pacemaker leads.^7^ Although generally successful, these cases carry a mild risk of increased pacing threshold and reduced ventricular sensing.^7^ Importantly, the risk of postprocedural lead dysfunction or late infection, although low, is not negligible, and it complicates future extraction planning. In such cases, the prosthetic valve frame often “jails” the lead against the annular ring or surgical prosthesis.

Although this may appear concerning, several reports have shown that jailing of RV pacing leads is frequently well tolerated, particularly when the lead remains undisturbed during valve deployment.^7^ Alternative strategies have been proposed for pacemaker-dependent patients, including preimplantation extraction of the transvenous lead with subsequent coronary sinus or epicardial lead placement. In the absence of such alternatives, procedural safety can be enhanced by using temporary pacing during deployment, particularly in fully pacemaker-dependent individuals. Our experience aligns with these insights, highlighting both the technical feasibility and need for individualized pacing strategies in transcatheter tricuspid ViR procedures involving indwelling leads. Lead function should be carefully monitored intra- and postprocedure, with backup pacing available. In our case, the pacing lead was successfully jailed without dislodgment or electrical compromise. The coaxial delivery of the valve, combined with the favorable implantation angle, facilitated safe expansion around the lead and preserved valve function.

Another important consideration during transcatheter ViR procedures for the tricuspid position is to confirm that the surgical ring is complete, as incomplete or open rings may increase the risk of paravalvular leak, embolization, or improper valve anchoring.^8^ Importantly, the 70:30 implantation ratio of the valve helped ensure sealing within the surgical valve/ring and minimized paravalvular leak without disturbing the pre-existing pacemaker lead. In addition, slow and controlled balloon inflation during deployment is essential to achieve optimal positioning, especially in the presence of a prior surgical ring. We recommend gradual inflation with coordinated communication among the interventional team to adjust valve orientation. Slight wire manipulation during inflation may be required to fine-tune the valve position and maintain the target 70:30 deployment ratio.

The use of the Edwards Inspiris Resilia valve in the pulmonary position remains relatively uncommon, with limited published experience. Although the Resilia valve has demonstrated favorable hemodynamic performance and durability in the aortic position, its use in transcatheter pulmonary ViV procedures has limited reports. In our case, the valve's flexible frame and anticalcification properties offered an advantageous profile for right-sided deployment. In our patient, we elected to implant the Edwards Inspiris Resilia valve in the pulmonary position after an unexpected storage failure rendered the intended Sapien valve unsuitable and no alternative 23-mm Sapien was available. Although this application was off-label, emerging data informed our decision. In a single-center retrospective cohort of 24 patients (median age: 26 years, 92% with prior ToF repair), the Inspiris Resilia demonstrated excellent early safety and efficacy at a median follow-up of 2.5 years, with no valve-related mortality, reintervention, or deterioration in gradient or pulmonary regurgitation.^9^ Conversely, in a group of 27 patients (mean age: 22 ± 15 years), trivial or mild regurgitation at discharge progressed to new prosthetic regurgitation in 48% of cases within an average of 16 months, with 22% developing moderate and 11% severe regurgitation; notably, ViV intervention was needed in 3 patients, and none of the conduit-implanted valves failed.^10^ Additionally, implantation within a native RV outflow tract was linked to higher failure rates compared with conduit placement.^10^ Further studies are still needed to assess the long-term performance and outcomes of Resilia valves in the pulmonary position, particularly in complex transcatheter interventions.

## Conclusions

This case represents the first reported instance of simultaneous transjugular ViR and ViV implantation in both the tricuspid and pulmonary positions. It highlights the feasibility, safety, and potential advantages of the transjugular approach in patients with challenging right heart anatomy and prior surgical repairs. The success of this procedure was facilitated by tailored planning, balloon-assisted sheath delivery, and strategic deployment technique. Transjugular intervention, when combined with tailored catheter strategies, may offer a safe and effective alternative in high-risk or inoperable patients. This experience contributes to the growing evidence supporting transcatheter solutions for complex right-sided valve disease and underscores the need for continued innovation and documentation in this evolving field. Looking ahead, such experiences may pave the way for broader adoption of dual-valve percutaneous strategies in the management of complex congenital heart disease, provided that further studies validate their long-term safety, durability, and clinical benefit.

**Visual Summary:**
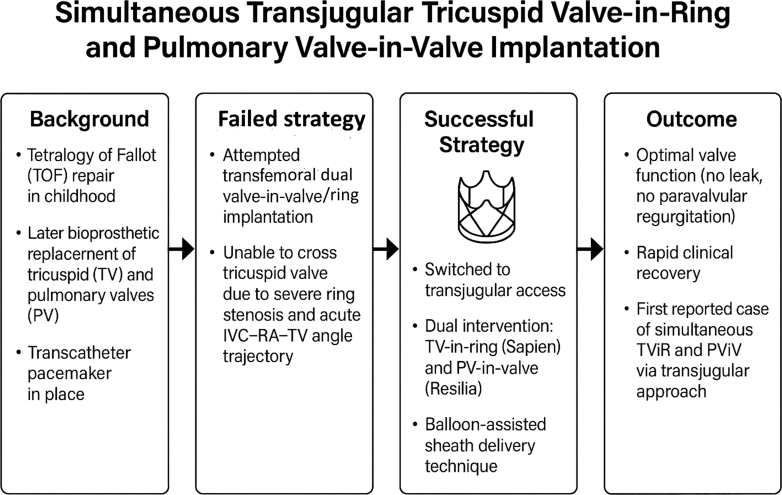
Case Presentation, Management, and Outcome IVC = inferior vena cava; RA = right atrium; TOF = tetralogy of fallot; TV = tricuspid valve; PV = pulmonary valve.

## Funding Support and Author Disclosures

The authors have reported that they have no relationships relevant to the contents of this paper to disclose.

## Appendix

Video 1 Sequential Demonstration of Transjugular Access Using a Long Peripheral Balloon to Facilitate Smooth Navigation and Support of the DrySeal Introducer System Through Tortuous Anatomy: Video 1 of 7

Video 2 Sequential Demonstration of Transjugular Access Using a Long Peripheral Balloon to Facilitate Smooth Navigation and Support of the DrySeal Introducer System Through Tortuous Anatomy: Video 2 of 7

Video 3 Sequential Demonstration of Transjugular Access Using a Long Peripheral Balloon to Facilitate Smooth Navigation and Support of the DrySeal Introducer System Through Tortuous Anatomy: Video 3 of 7

Video 4 Sequential Demonstration of Transjugular Access Using a Long Peripheral Balloon to Facilitate Smooth Navigation and Support of the DrySeal Introducer System Through Tortuous Anatomy: Video 4 of 7

Video 5 Sequential Demonstration of Transjugular Access Using a Long Peripheral Balloon to Facilitate Smooth Navigation and Support of the DrySeal Introducer System Through Tortuous Anatomy: Video 5 of 7

Video 6 Sequential Demonstration of Transjugular Access Using a Long Peripheral Balloon to Facilitate Smooth Navigation and Support of the DrySeal Introducer System Through Tortuous Anatomy: Video 6 of 7

Video 7 Sequential Demonstration of Transjugular Access Using a Long Peripheral Balloon to Facilitate Smooth Navigation and Support of the DrySeal Introducer System Through Tortuous Anatomy: Video 7 of 7

Video 8 Deployment of the Edwards Sapien Resilia Valve Within the Bioprosthetic Pulmonary Position, Showing Precise Positioning and Expansion

Video 9 Implantation of the Edwards Sapien S3 Valve in the Tricuspid Ring Position, Illustrating Optimal Alignment and Successful Valve-in-Ring Deployment

Video 10 Transesophageal Echocardiogram Showing No Regurgitation in the Tricuspid Valve After Pulmonary Valve-in-Valve and Tricuspid Valve-in-Ring Deployment, and No Paravalvular Leak

Video 11 Three-Dimensional Transesophegeal Echocardiogram Showing No Regurgitation in the Tricuspid Valve After Tricuspid Valve-in-Ring Implantation
